# *CYP4F2* and *CYP3A5* gene polymorphisms and lung cancer in Chinese Han population

**DOI:** 10.1007/s10238-020-00631-6

**Published:** 2020-04-30

**Authors:** Ruiqing He, Meng Li, Anqi Li, Wenhui Dang, Tian Yang, Jing Li, Ning Zhang, Tianbo Jin, Mingwei Chen

**Affiliations:** 1 The Department of Respiratory and Critical Care Medicine of the First Affiliated Hospital of Xi’an Jiaotong University, 277 West Yanta Road, Xi’an, 710061 Shaanxi China; 2 Shaanxi Provincial Research Center for the Project of Prevention and Treatment of Respiratory Diseases, 1# Xin Wang Road, Xi’an, 710021 Shaanxi China; 3 Ministry of Education Key Laboratory of Resource Biology and Biotechnology in Western China (Northwest University), #229 North Taibai Road, Xi’an, 710069 Shaanxi China

**Keywords:** Gene polymorphisms, *CYP3A5*, *CYP4F2*, Lung cancer

## Abstract

This study aimed to explore whether the polymorphisms of *CYP4F2* and *CYP3A5* are correlated with the risk of lung cancer development. A case–control study was conducted among 510 patients with pathologically confirmed lung cancer as the case group and 504 healthy individuals as the control group. Four single-nucleotide polymorphisms of the *CYP4F2* and *CYP3A5* genes were genotyped, and their correlations with the risk of lung cancer were examined using Chi-square test and logistic regression analysis. Stratified analysis found that the rs3093105 and rs3093106 loci of *CYP4F2* gene were significantly associated with lower risk of lung cancer (*P* = 0.012, OR 0.64, 95% CI 0.45–0.91). The correlation was related to patients’ age and sex and pathological type of lung cancer. Similarly, the rs10242455 loci of *CYP3A5* gene showed a statistical significance between the case group and the control group (*P* = 0.018, OR 0.71, 95% CI 0.53–0.94), which also was associated with reduced risk of squamous cell lung cancer in the dominant and additive models (dominant: OR 0.66, 95% CI 0.46–0.94, *P* = 0.021; additive: OR 0.71, 95% CI 0.53–0.95, *P* = 0.023). *CYP4F2* and *CYP3A5* gene polymorphisms are associated with the reduced risk of non-small cell lung cancer, and its correlation is related to patients’ age and sex and pathological type of lung cancer.

## Introduction

Lung cancer is one of the most common malignant tumors [1]. With the increase in detection rate of lung cancer and the aggravation of environmental pollution, morbidity and mortality of lung cancer are increasing year by year [2]. At present, lung cancer has become the leading cause of cancer death worldwide in both sexes combined (18.4% of the total cancer deaths) [3, 4]. According to statistics, in 2012, 1.8 million new cases of lung cancer occurred, accounting for about 13% of new cancer [1, 4, 5]. Epidemiological studies have demonstrated that lung cancer diagnoses are associated with smoking in highly developed countries [6], but only 10–15% of smokers develop lung cancer in all smokers, and the morbidity of lung cancer varies in different genders, races, and regions. Studies show that lung cancer has the highest morbidity in North America, East Asia, the Middle East, and the Southern Europe, while female lung cancer rates are highest in North America and Southern Europe [5, 7]. In addition, many studies have shown that the development, metastasis, and prognosis of lung cancer are related to many gene mutations, such as EGFR, KRAS, and BRAF [8, 9]. The above researches indicated that lung cancer is not only caused by environmental factors, genetic factors cannot be ignored in the occurrence and development of lung cancer.

Cytochrome *P450* (*CYP450*), belonging to ω-hydroxylase, participates in the metabolism of many endogenous substances and exogenous compounds, including fatty acids, docosahexenoic acid, and vitamin D, and of a wide variety of carcinogens and anticancer drugs [10, 11]. These reactive metabolites would interact with DNA, thereby causing altered gene expression or function, and eventually carcinogenesis [12]. Therefore, *CYP450* may influence tumor genesis and progression. *CYP4F2* and *CYP3A5* are members of the *CYP450* family. A recent study shows that the expression of *CYP4F2* is closely related to hepatocellular carcinoma cells, which may contribute to tumor progression [10]. Relative studies demonstrate that 20-HETE (*CYP4F2*-related products) was associated with the growth of tumors in mouse non-small cell lung cancer cell lines [13]. Another study confirmed that *CYP3A5* was associated with lung cancer in the population of Taiwan, China [14]. However, little research has been done about the association between *CYP4F2* and *CYP3A5* gene polymorphisms and lung cancer in the Chinese Han population of mainland China.

Therefore, in this study, four SNP locus in *CYP4F2* and *CYP3A5* genes were analyzed to explore the association between the polymorphisms of *CYP4F2* and *CYP3A5* genes and the risk for lung cancer.

## Materials and methods

### Subject recruitment and sample collection

A case–control study, involving 510 lung cancer patients as the case group and 504 healthy individuals as the control group, was conducted at the First Affiliated Hospital of Xi’an Jiaotong University, Shaanxi, China. All included patients had recently been diagnosed and pathologically confirmed primary lung cancer. The subjects in the control group were recruited from the Health Checkup Center of the First Affiliated Hospital of Xi’an Jiaotong University, who take health examination annually and have no histories of cancers and no chronic or serious endocrine or metabolic nutritional diseases. Patients were ascertained to be free from any acute or chronic pathology. Blood samples from the patients with lung cancer were collected before initiation of chemotherapy or radiotherapy. All of the participants were genetically unrelated ethnic Han Chinese and agree to participate in the present study. The protocols for this study were approved by the Ethical Committees of both the First Affiliated Hospital of Xi’an Jiaotong University and Northwest University.

Five milliliters of whole blood was collected from each subject into tubes containing ethylenediaminetetraacetic acid at the time of initial diagnosis. After centrifugation, the samples were stored at − 80 °C until further use. The characteristics of all study participants are summarized in Table 1.

**Table 1:** Characteristics of case group and control group in the study

Variable	Case (*n* = 510)	Control (*n* = 504)	*P* value
Sex			0.911
Female	126 (24.7)	123 (24.4)	
Male	384 (75.3)	381 (75.6)	
Age, years (mean ± SD)	58.08 ± 10.548	57.27 ± 10.852	0.227
TNM stage			
I–II	129 (25.3)		
III–IV	248 (48.6)		
Miss	133 (26.1)		
Pathological types			
SCC	169 (33.1)		
AC	161 (31.6)		
SCLC	97 (19.0)		
Others	22 (4.3)		
Miss	61 (12.0)		

*SCLC* small cell lung cancer

### SNPs selection and primer design

Based on GWAS studies of tumors and reports in related literature, four SNP loci of *CYP4F2* and *CYP3A5* genes were selected. All loci met the criterion that the minimum allele frequency was more than 5% in HapMap Chinese Han Beijing population. Primers were designed according to ASSAY Design SUITE V2.0 (https://agenacx.com/online-tools). (All primers were designed according to the sequence of forward strand from dbSNP Database.)

### DNA purity detection and genotyping

DNA was extracted by whole blood genome DNA purification kit (Xi’an GoldMag Biological Company). The concentration and purity of DNA were detected by Nanodrop Lite ultraviolet spectrophotometer (Thermo Scientific, Waltham, Massachusetts, USA). Genotyping of all SNPs was performed on Mass ARRAY iPLEX (Agena Bioscience, San Diego, CA, USA) platform using matrix-assisted laser desorption ionization time of flight (MALDI-TOF) mass spectrometer. The results were output by Agena Bioscience TYPER 4.0 software.

### Statistical analysis

Microsoft Excel (Microsoft, Redmond, WA) and SPSS software(version 19.0, SPSS, Chicago, IL) were used for statistical analysis. Chi-square test was taken to compare the distribution of frequency of suspicious confounding factors (age, sex, etc.) in cases and control groups, to determine the comparability between the two groups. Hardy–Weinberg equilibrium test (HWE) was performed on all SNP frequencies in the control group by Chi-square test. Fisher's exact test was used to compare the allele and genotype frequencies of each locus in two groups. We used logistic regression analysis to assess the association between each SNP and the risk of lung cancer and risk for lung cancer in different genetic models (additive, dominant, recessive models), while conducting management considering age and gender. Logistic regression analysis was also used to calculate odds ratios (ORs) and 95% confidence intervals (CIs). In the comparisons above, a two-sided *P* value < 0.05 was considered statistically significant. According to the stratification of age, gender, and pathological types of lung cancer, the correlation between SNP sites and lung cancer risk in different stratified populations was evaluated. The specific method was the same as the above.

## Results

### Population characteristics

510 cases of lung cancer were included in this study. The average age was 58.08 (± 10.548) years old in the cases, of which 75.3% were males and 24.7% females. 504 cases were included in the control group, with an average age of 57.27 (± 10.852) years old, of which 75.6% were males and 24.4% females. Chi-square test showed that there was no significant difference in age and sex between the case group and the control group (age: *P* = 0.227, gender: *P* = 0.911) (Table 1).

### SNP and the risk of lung cancer

Basic information of four SNPs loci in *CYP4F2* and *CYP3A5* genes is shown in (Table 2). All SNPs loci were in accordance with Hardy–Weinberg equilibrium (HWE) assessed by Chi-square test and Fisher’s exact test of SPSS software. The distributions of allele frequencies between the case group and the control group were compared by Chi-square test of Plink software. The results showed that the *P* values of all SNPs loci in the whole population were greater than 0.05 in allele model, which demonstrated that there was no significant difference between the two groups in the whole population.

**Table 2:** SNP in CYP4F2 and CYP3A5 gene

SNP ID	Genes	Band	Position	Role	Alleles A/B	*p*-HWE	*P*^a^	OR (95% CI)
rs10242455	CYP3A5	7	99642556	Intron variant	G/A	0.396	0.403	0.92 (0.76–1.12)
rs2108622	CYP4F2	19	15879621	Missense	T/C	0.664	0.349	0.91 (0.75–1.11)
rs3093106	CYP4F2	19	15897447	Synonymous codon	C/T	1.000	0.128	0.80 (0.61–1.07)
rs3093105	CYP4F2	19	15897578	Missense	C/A	1.000	0.112	0.80 (0.60–1.05)

*SNP* single-nucleotide polymorphism, *OR* odds ratio, *95% CI* 95% confidence interval, *HWE* Hardy–Weinberg equilibrium

^a^Two-sided Chi-square tests; *p ≤ 0.05 indicates statistical significance

From the analysis above, it can be concluded that four SNPs loci in *CYP4F2* and *CYP3A5* genes have no significant correlation with risk for lung cancer in the case group and the control group in allele model, so stratified analysis was carried out in the aspect of age, sex, and pathological type of lung cancer. Stratified analysis found that three of the four selected SNP loci were significantly associated with lowered risk of lung cancer in allele model, namely rs3093106 (*CYP4F2*), rs3093105 (*CYP4F2*), and rs10242455 (*CYP3A5*), among which rs3093106 and rs3093105 were significantly different between the two groups in the subjects of older than 58 years old (rs3093105: *P* = 0.023, OR 0.59, 95% CI 0.37–0.93; rs3093106: *P* = 0.029, OR 0.60, 95% CI 0.38–0.94), lung adenocarcinoma (rs3093105, *P* = 0.023, OR 0.59, 95% CI 0.37*–*0.93; rs3093106: *P* = 0.025; OR 0.60, 95% CI 0.38–0.94) and male patients (rs3093105, *P* = 0.017, OR 0.68, 95% CI 0.49–0.93; rs3093106: *P* = 0.020, OR 0.68, 95% CI 0.50–0.94). Rs10242455 in *CYP3A5* gene showed significant difference between the two groups in lung squamous cell carcinoma (rs10242455: *P* = 0.018, OR 0.71, 95% CI 0.53–0.94) (Tables 3, 4).

**Table 3:** Association between CYP4F2 gene polymorphism and lung cancer under different stratification analyses (adjusted by sex and age)

SNP ID	Years ≥ 58		Years < 58		Male		Female		Adenocarcinoma		SCC		SCLC	
	OR (95% CI)	*P*^a^	OR (95% CI)	*P*^a^	OR (95% CI)	*P*^a^	OR (95% CI)	*P*^a^	OR (95% CI)	*P*^a^	OR (95% CI)	*P*^a^	OR (95% CI)	*P*^a^
*rs3093105*														
Alleles														
C/A	0.59 (0.37–0.93)	0.023*	1.02 (0.68–1.52)	0.942	0.68 (0.49–0.93)	0.017*	1.41 (0.77–2.58)	0.260	0.59 (0.37–0.93)	0.023	1.01 (0.69–1.48)	0.951	0.75 (0.45–1.26)	0.277
Genotype														
A/A	1	0.073	1	0.995	1	0.049*	1	0.478	1	0.105	1	0.641	1	0.820
C/A	0.66 (0.43–1.02)		1.00 (0.63–1.57)		0.71 (0.50–1.01)		1.27 (0.66–2.45)		0.60 (0.36–0.99)		1.01 (0.66–1.55)		0.84 (0.48–1.46)	
C/C	0.21 (0.02–1.91)		1.08 (0.22–5.44)		0.26 (0.05–1.27)		31.989E+09 (0.00–∞)		0.45 (0.05–3.74)		0.36 (0.04–3.01)		2.949E−09 (0–∞)	
Dominant														
A/A		0.035*		0.100	1	0.029*	1	0.334	1	0.034*	1	0.880	1	0.389
C/A–C/C	0.63 (0.41–0.97)		1.00 (0.64–1.56)		0.68 (0.48–0.96)		1.38 (0.72–2.64)		0.59 (0.36–0.96)		0.97 (0.64–1.47)		0.78 (0.45–1.36)	
Recessive														
A/A–C/A	1	0.191	1	0.923	1	0.114	1	0.100	1	0.514	1	0.346	1	0.999
C/C	0.23 (0.03–2.08)		1.08 (0.22–5.43)		0.28 (0.06–1.36)		1.887E+09 (0–inf)		0.50 (0.06–4.09)		0.36 (0.04–2.99)		3.061E−09 (0–∞)	
Additive														
A/A	0.63 (0.42–0.94)	0.023*	1.01 (0.67–1.51)	0.980	0.67 (0.48–0.93)	0.016*	1.47 (0.80–2.71)	0.220	0.61 (0.38–0.97)	0.035*	0.93 (0.63–1.37)	0.704	0.75 (0.44–1.27)	0.280
C/A														
C/C														
*rs3093106*														
Alleles														
C/T	0.60 (0.38–0.94)	0.029*	1.02 (0.68–1.52)	0.942	0.68 (0.50–0.94)	0.020*	1.41 (0.77–2.58)	0.260	0.60 (0.38–0.94)	0.025*	1.02 (0.70–1.49)	1.02	0.76 (0.45–1.27)	0.293
Genotype														
T/T	1	0.089		0.995	1	0.060	1	0.478	1	0.114	1	0.636	1	0.839
C/T	0.68 (0.44–1.04)		1.00 (0.63–1.57)		0.72 (0.51–1.03)		1.27 (0.66–2.45)		0.60 (0.37–1.00)		1.03 (0.67–1.57)		0.85 (0.48–1.47)	
C/C	0.21 (0.02–1.92)		1.08 (0.22–5.44)		0.26 (0.05–1.27)		31.989E+09 (0.00–∞)		0.45 (0.05–3.74)		0.37 (0.04–3.02)		2.956E−09 (0–∞)	
Dominant														
T/T	1	0.045*		0.100	1	0.036*	1	0.334	1	0.038*	1	0.940	1	0.408
C/T–C/C	0.65 (0.42–0.99)		1.00 (0.64–1.56)		0.69 (0.48–0.9)		1.38 (0.72–2.64)		0.59 (0.36–0.97)		0.98 (0.65–1.50)		0.79 (0.45–1.38)	
Recessive														
T/T–C/T	1	0.190	1	0.923	1	0.114	1	0.100	1	0.514	1	0.346	1	0.999
C/C	0.23 (0.03–2.08)		1.08 (0.22–5.43)		0.28 (0.06–1.36)		1.887E+09 (0–inf)		0.50 (0.06–4.09)		0.36 (0.04–2.99)		3.061E−09 (0–∞)	
Additive														
T/T	0.64 (0.43–0.96)	0.029*	1.01 (0.67–1.51)	0.980	0.68 (0.49–0.94)	0.019*	1.47 (0.80–2.71)	0.220	0.61 (0.39–0.98)	0.039*	0.94 (0.64–1.39)	0.757	0.75 (0.45–1.28)	0.294
C/T														
C/C														

*SNP* single-nucleotide polymorphism, *OR* odds ratio, *95% CI* 95% confidence interval, *SCC* squamous cell carcinoma, *SCLC* small cell lung cancer

^a^Two-sided Chi-square tests; *p ≤ 0.05 indicates statistical significance

**Table 4:** Association between CYP3A5 gene polymorphism and lung cancer under different pathological types (adjusted by sex and age)

SNP ID	SCC		Adenocarcinoma		SCLC	
	OR (95% CI)	*P*^a^	OR (95% CI)	*P*^a^	OR (95% CI)	*P*^a^
*rs10242455*						
Alleles						
G/A	0.71 (0.53–0.94)	0.018*	1.26 (0.96–1.64)	0.091	0.82 (0.58–1.16)	0.261
Genotype						
A/A	1	0.064	1	0.252	1	0.317
G/A	0.67 (0.46–0.98)		1.29 (0.88–1.88)		0.70 (0.44–1.11)	
G/G	0.57 (0.27–1.19)		1.58 (0.83–3.02)		0.85 (0.37–1.91)	
Dominant						
A/A	1	0.021*	1	0.126	1	0.145
G/A–G/G	0.66 (0.46–0.94)		1.33 (0.92–1.92)		0.72 (0.46–1.12)	
Recessive						
A/A–G/ A	1	0.274	1	0.290	1	0.970
G/G	0.67 (0.32–1.38)		1.40 (0.75–2.58)		0.98 (0.44–2.18)	
Additive						
A/A	0.71 (0.53–0.95)	0.023*	1.27 (0.96–1.68)	0.097	0.81 (0.57–1.16)	0.248
G/A						
G/G						

*SNP* single-nucleotide polymorphism, *OR* odds ratio, *95% CI* 95% confidence interval, *SCC* squamous cell carcinoma, *SCLC* small cell lung cancer

^a^Two-sided Chi-square tests; *p ≤ 0.05 indicates statistical significance

In addition, three selected SNPs loci were analyzed in different populations and different genetic models through logistic regression analysis. The results showed that the rs3093105 and rs3093106 were linked with reduced risk of lung cancer in the dominant model and additive model of lung adenocarcinoma, male patients and patients older than 58 years old. After adjusting for age and gender, the correlation was still observed (Table 3). The rs10242455 was associated with lowered risk of lung squamous cell carcinoma in the dominant model and additive model; after adjusting for age and gender, the correlation was still observed (Table 4).

**Fig. 1:**
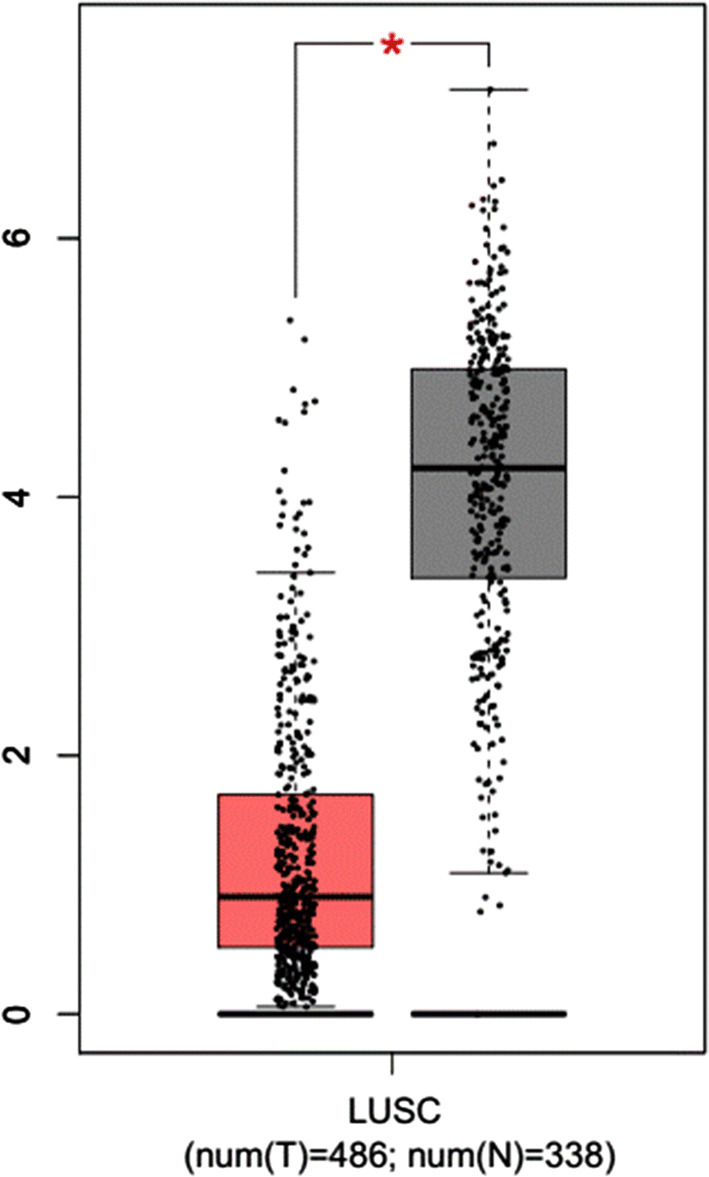
Expression of CYP3A5 gene in lung squamous cell carcinoma (*n* = 486) and para-tumor tissues (*n* = 338) from GEPIA database. The *Y*-axis is the log-scale of log _2_(TPM + 1) (*TPM* Transcripts Per Million). The box plots show the interquartile range (IQR), median (bar in box), tissues. CYP3A5 expression is significantly lower in lung squamous cell carcinoma (*p < 0.01)

**Fig. 2:**
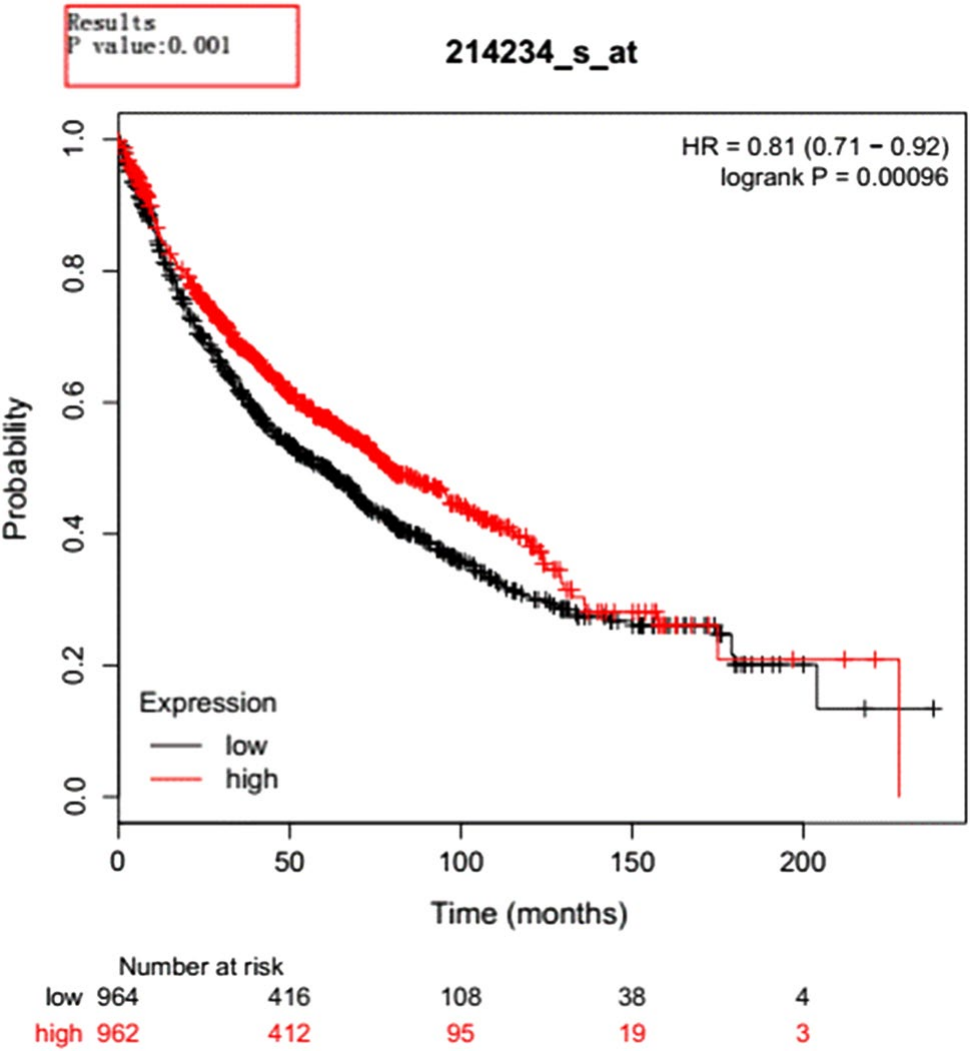
Relationship between CYP3A5 gene and prognosis of lung squamous cell carcinoma. The *Y*-axis is survival rate. Red line represents low expression of CYP3A5 gene, and black line represents high expression of CYP3A5 gene in lung squamous carcinoma people; *P* value < 0.05 was considered statistically significant; *HR* Hazard ratio; the numbers at the bottom indicate the number of people still alive at the different survival time (color figure online)

In addition, through the analysis of TCGA database, GEPIA database (http://gepia.cancer-pku.cn/), Kaplan–Meier plotter database (http://kmplot.com/), the correlation between expression and prognosis was analyzed. It was found that the expression of *CYP3A5* gene in cancer tissues is lower than that in para-tumor tissues (Fig. 1), and there was a worse prognosis in lung cancer patients with lower expression (Fig. 2).

## Discussion

This study suggests that *CYP3A5* and *CYP4F2* were associated with reduced risk of NSCLC. This was related to age, sex, and pathological type of lung cancer.

*CYP4F2* is a member of the *CYP4F* family. Several studies have revealed marked mRNA up-regulation of genes encoding CYP4 enzymes in thyroid, breast, colon, and ovarian cancers. Alexanian et al. [15] confirmed that the levels of *CYP4F2* and 20-HETE in ovarian cancer tissues were higher than those in normal control group. However, up to now, the correlation between *CYP4F* gene and lung cancer has not been reported. Our study has been the first to report that there is a significant correlation between *CYP4F2* gene polymorphisms and lung cancer in Chinese Han population, and this is associated with lowered risk of lung cancer in people older than 58 years old, lung adenocarcinoma and men. Similarly, Ankit et al. confirmed that the expression of *CYP4F2* was increased in pancreatic ductal carcinoma, and the expression of *CYP4F2* was negatively correlated with age and higher in males [16]. This is similar to the conclusion of the present study. In addition, many studies have confirmed that *CYP4F2* was closely related to the metabolism of 20-hydroxyethyl hexadecanoic acid (20-HETE) [17–19]. In the past decade, 20-HETE has been recognized as a key conditioning agent of cancer progression, which can induce cell proliferation in vitro by stimulating the formation of reactive oxygen species and the production of vascular endothelial growth factor. Previous studies have shown that 20-HETE antagonists (WIT002) can inhibit the proliferation of renal adenocarcinoma [20]. Similarly, two studies have demonstrated that HET0016 (20-HETE antagonist) can inhibit the growth of tumors in non-small cell lung cancer cell lines and of human glioma [13, 21]. We hypothesize that the effect of *CYP4F2* gene polymorphisms on the risk for lung cancer may be related to the metabolism of 20-HETE and then affect the growth of cancer cells by regulating the signal pathway of vascular endothelial growth factor. However, further experiments are needed to confirm this.

*CYP3A5* is an important member of the *CYP3A* family. It participates in the catalytic oxidation of many exogenous substances, including toxins, carcinogens, the metabolism, and clearance of some drugs [1]. Studies have shown that *CYP3A5* plays an important role in the development of acute and chronic leukemia, colorectal cancer, and esophageal cancer [22–25]. Islam et al. [26] reported that *CYP3A5* was a risk factor of lung cancer in Bangladeshi population. Interestingly, we found that *CYP3A5* was a protective factor of NSCLC in Chinese Han population, which may be related to racial differences. Similarly, in a study of Taiwanese of China, *CYP3A5* has been confirmed to play a protective role in the development of lung cancer [14]. Also, Feng et al. indicated that *CYP3A5* plays a protective role in the occurrence and metastasis of hepatocellular carcinoma. At the same time, they also confirmed that *CYP3A5* over-expression in hepatocellular carcinoma cells inhibits the metastasis and invasion of cancer cells in vivo and in vitro, via manipulating ROS/mTORC2/p-AKT (S473) signaling pathway and limiting MMP2/9 function [25, 27]. Research has found that a SNP within intron-3 (CYP3A5*3) results in aberrant mRNA splicing and a pronounced reduction in protein synthesis [28]. Likewise, rs10242455 belongs to intron variants in CYP3A5 gene. So, we suspect that CYP3A5 may affect ROS/mTORC2/p-AKT (S473) signaling pathway and limiting MMP2/9 function by affecting mRNA splicing and protein synthesis, thereby affecting the occurrence of lung cancer.

In addition, we found that *CYP3A5* gene was low expressed in lung squamous cell carcinomas, and the survival rate was lower among the lung cancer patients with low expression. Similarly, Tingdong suggests that the lower the expression of *CYP3A5*, the worse the prognosis in hepatocellular carcinoma patients [25]. Another study in Chinese population showed that *CYP3A5* gene is closely related to the prognosis of patients with non-small cell lung cancer undergoing chemotherapy and surgical treatment [1]. This is similar to our conclusion. Besides, two recent studies indicated that *CYP3A5* gene participates in the metabolism of docetaxel and sunitinib. Different genotypes respond differently to drug dosage requirements and drug toxicity [29, 30]. This suggests that *CYP3A5* gene may be related not only to the risk and prognosis of lung cancer, but also to the treatment and drug selection of lung cancer. It may be a predictor of the occurrence, development, and prognosis of lung cancer, but it needs a larger sample of research to further confirm the findings.

Our research confirms that *CYP4F2* and *CYP3A5* gene polymorphisms are associated with the risk of lung cancer. We believe that our results will encourage more people using larger sample sizes to further confirm the relationship between *CYP4F2* and *CYP3A5* genes and lung cancer, as well as their specific mechanisms in the occurrence and development of lung cancer in the future studies. But there are still limitations for our study. First, the treatment and survival time of lung cancer patients did not take into consideration. Second, we did not collect the smoking data of the samples in our study, and further study is needed to improve the deficiencies of this research.

## Conclusion

This study found that *CYP4F2* and *CYP3A5* gene polymorphisms were associated with the risk of NSCLC.
